# Supplementary material for: Nitrile glove composition and performance—Substandard properties and inaccurate packaging information
Source: PLoS One. 2024 Oct 31;19(10):e0312891. doi: 10.1371/journal.pone.0312891 (PMC11527158; doi:10.1371/journal.pone.0312891)
Supplement: S1 File — (PDF) [file pone.0312891.s001.pdf]

Sample: American Nitrile (green)

13C SSNMR (quantitative multicp)

600MHz Bruke AVIIIHD; 3.2mm HXY

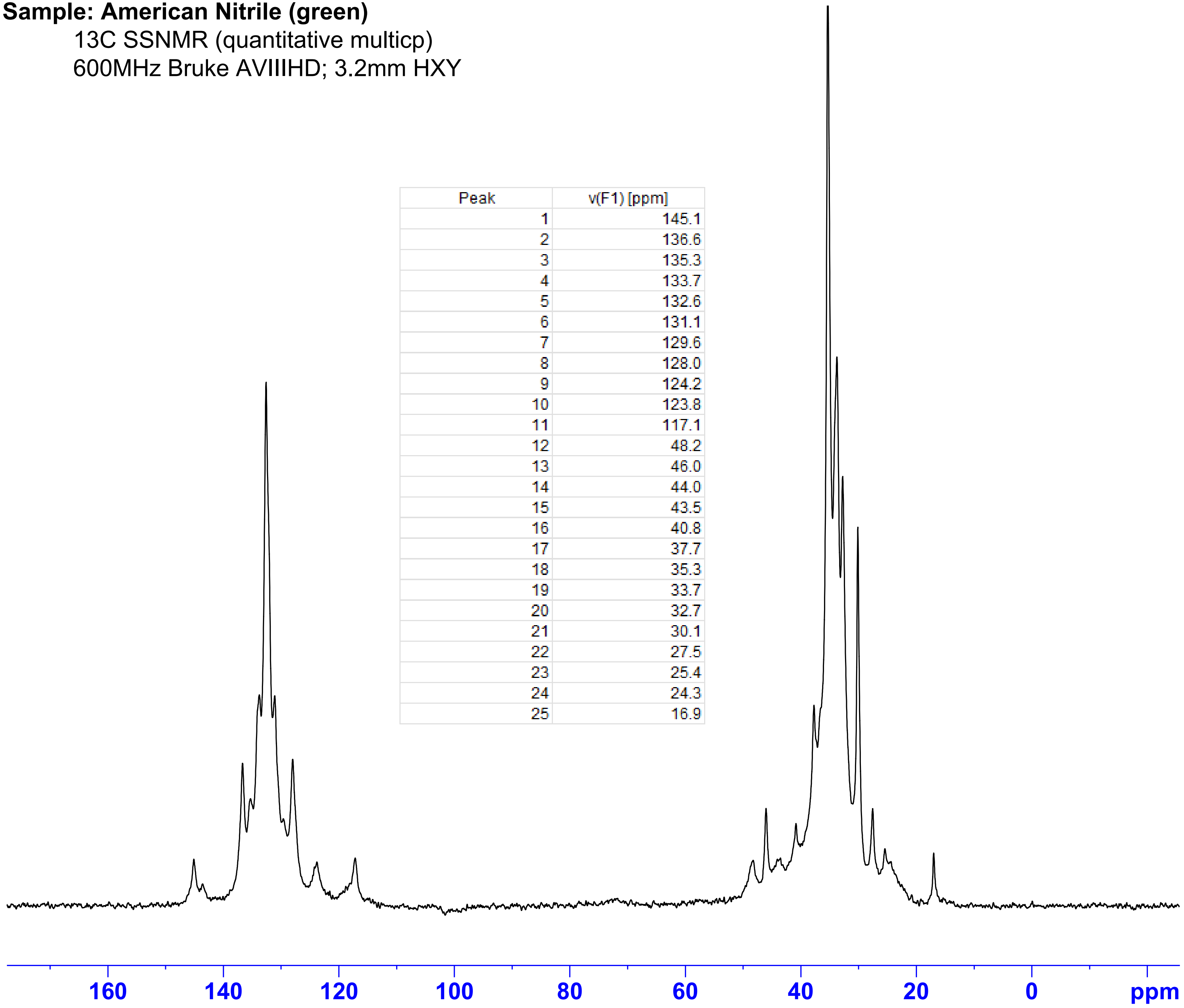

Peak	ν(F1) [ppm]
1	145.1
2	136.6
3	135.3
4	133.7
5	132.6
6	131.1
7	129.6
8	128.0
9	124.2
10	123.8
11	117.1
12	48.2
13	46.0
14	44.0
15	43.5
16	40.8
17	37.7
18	35.3
19	33.7
20	32.7
21	30.1
22	27.5
23	25.4
24	24.3
25	16.9

```
Current Data Parameters
NAME      HXY_13C_Vodovotz
EXPNO                    6
PROCNO                   1

F2 - Acquisition Parameters
Date_             20230503
Time                  5.30 h
INSTRUM              spect
PROBHD    H127000_0013 (
PULPROG        multicp_tlw
TD                    4096
SOLVENT
NS                   10240
DS                       8
SWH              59523.809 Hz
FIDRES           29.064360 Hz
AQ               0.0344064 sec
RG                  191.56
DW                   8.400 usec
DE                    6.50 usec
TE                       0 K
D1              3.00000000 sec
D3              0.00003400 sec
D11             0.40000001 sec
D13             0.00004400 sec
L0                       9
L31                  20000
ZGOPTNS
SFO1           150.9489952 MHz
NUC1                   13C
P1                    5.00 usec
P2                   10.00 usec
P5                  100.00 usec
P6                  100.00 usec
PLW1           44.00000000 W
PLW11          44.00000000 W
SFO2           600.2555479 MHz
NUC2                    1H
CPDPRG[2       spinal64_13
P3                    2.50 usec
PCPD2                 4.80 usec
PLW2          112.00000000 W
PLW3           81.00000000 W
PLW13         112.00000000 W
am2       90 %

F2 - Processing parameters
SI                   16384
SF             150.9338167 MHz
WDW                     EM
SSB                      0
LB                   20.00 Hz
GB                       0
PC                    0.20
```

Sample: American Nitrile (purple)

13C SSNMR (quantitative multicp)

600MHz Bruke AVIIIHD; 3.2mm HXY

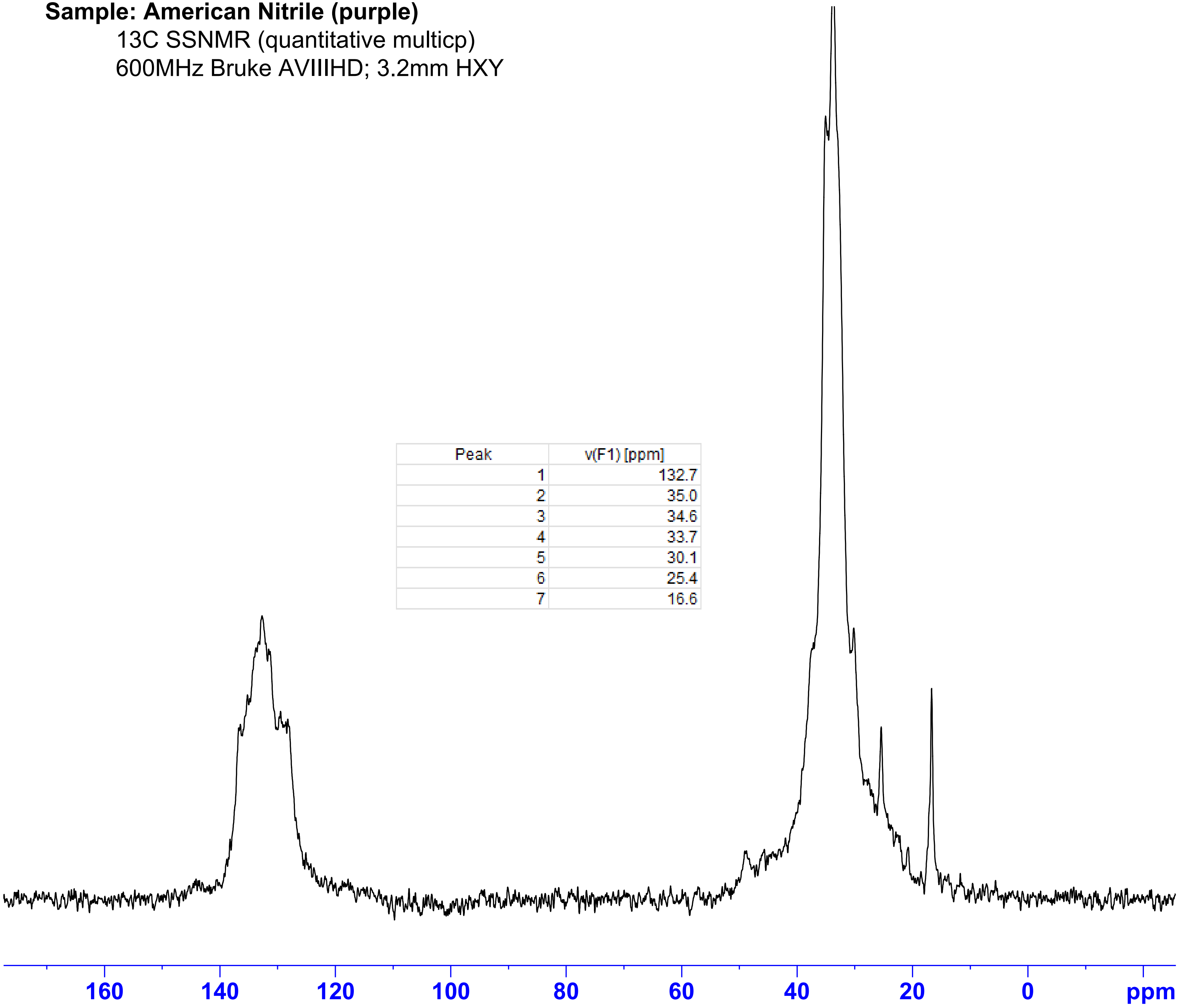

Peak	v(F1) [ppm]
1	132.7
2	35.0
3	34.6
4	33.7
5	30.1
6	25.4
7	16.6

Current Data Parameters

NAME	HXY_13C_Vodovotz
EXPNO	8
PROCNO	1

F2 - Acquisition Parameters

Date_	20230505
Time	7.49 h
INSTRUM	spect
PROBHD	H127000_0013 (
PULPROG	multicp_tlw
TD	4096
SOLVENT	
NS	10240
DS	8
SWH	59523.809 Hz
FIDRES	29.064360 Hz
AQ	0.0344064 sec
RG	191.56
DW	8.400 usec
DE	6.50 usec
TE	0 K
D1	3.00000000 sec
D3	0.00003400 sec
D11	0.40000001 sec
D13	0.00004400 sec
L0	9
L31	20000
ZGOPTNS	
SFO1	150.9489952 MHz
NUC1	13C
P1	5.00 usec
P2	10.00 usec
P5	100.00 usec
P6	100.00 usec
PLW1	44.00000000 W
PLW11	44.00000000 W
SFO2	600.2555479 MHz
NUC2	1H
CPDPRG[2	spinal64_13
P3	2.50 usec
PCPD2	4.80 usec
PLW2	112.00000000 W
PLW3	81.00000000 W
PLW13	112.00000000 W
am2	90 %

F2 - Processing parameters

SI	16384
SF	150.9338167 MHz
WDW	EM
SSB	0
LB	20.00 Hz
GB	0
PC	0.20

Sample: American Nitrile (slate)

13C SSNMR (quantitative multicp)

600MHz Bruke AVIIIHD; 3.2mm HXY

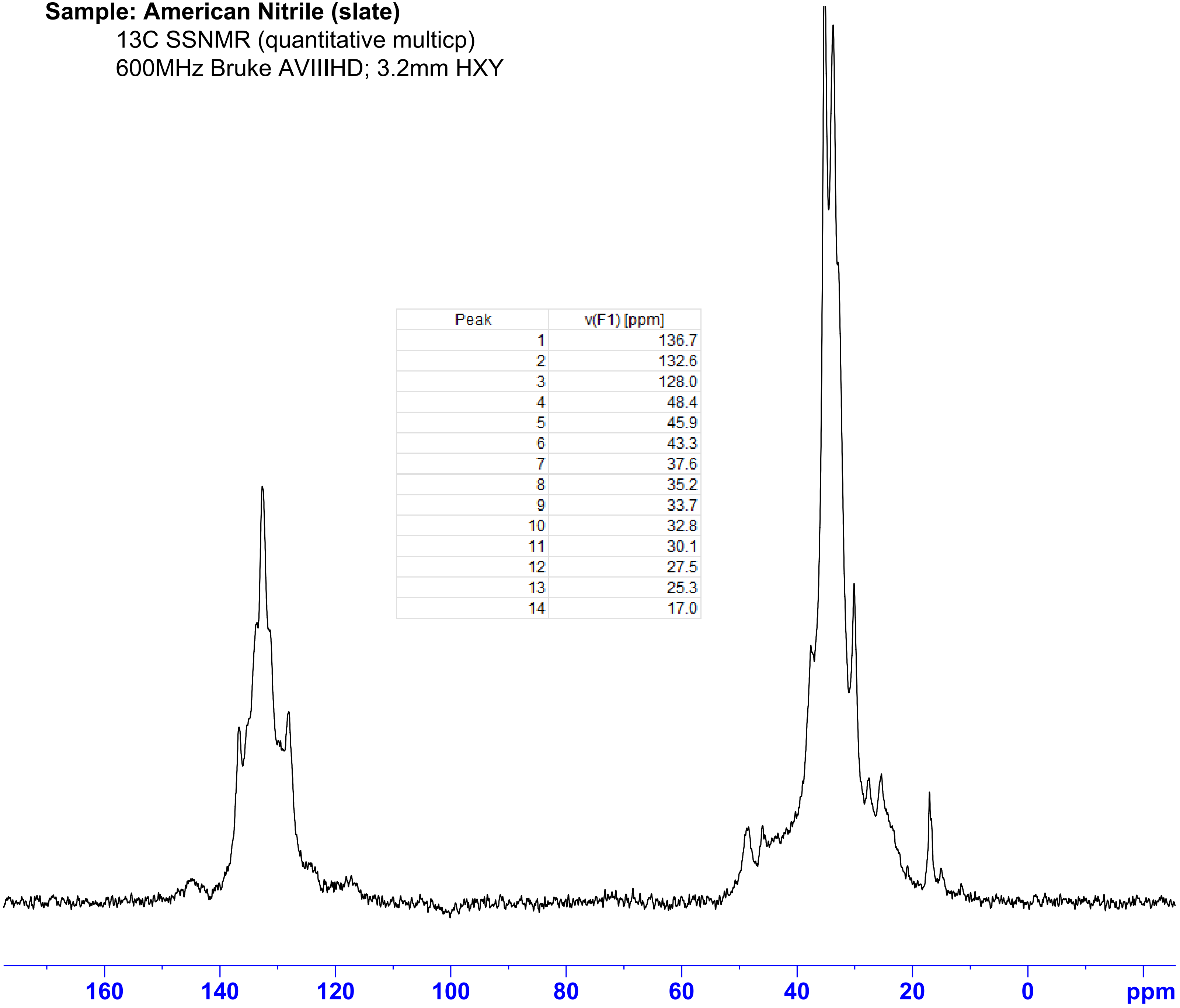

Peak	ν(F1) [ppm]
1	136.7
2	132.6
3	128.0
4	48.4
5	45.9
6	43.3
7	37.6
8	35.2
9	33.7
10	32.8
11	30.1
12	27.5
13	25.3
14	17.0

```
Current Data Parameters
NAME       HXY_13C_Vodovotz
EXPNO                     7
PROCNO                    1

F2 - Acquisition Parameters
Date_              20230504
Time                 5.38 h
INSTRUM               spect
PROBHD     H127000_0013 (
PULPROG         multicp_tlw
TD                     4096
SOLVENT
NS                    10240
DS                        8
SWH               59523.809 Hz
FIDRES            29.064360 Hz
AQ                0.0344064 sec
RG                   191.56
DW                    8.400 usec
DE                     6.50 usec
TE                        0 K
D1               3.00000000 sec
D3               0.00003400 sec
D11              0.40000001 sec
D13              0.00004400 sec
L0                        9
L31                   20000
ZGOPTNS
SFO1            150.9489952 MHz
NUC1                    13C
P1                     5.00 usec
P2                    10.00 usec
P5                   100.00 usec
P6                   100.00 usec
PLW1            44.00000000 W
PLW11           44.00000000 W
SFO2            600.2555479 MHz
NUC2                     1H
CPDPRG[2        spinal64_13
P3                     2.50 usec
PCPD2                  4.80 usec
PLW2           112.00000000 W
PLW3            81.00000000 W
PLW13          112.00000000 W
am2        90 %

F2 - Processing parameters
SI                    16384
SF              150.9338167 MHz
WDW                      EM
SSB                       0
LB                    20.00 Hz
GB                        0
PC                     0.20
```

Sample: N-Dex Plus

13C SSNMR (quantitative multicp)

600MHz Bruke AVIIIHD; 3.2mm HXY

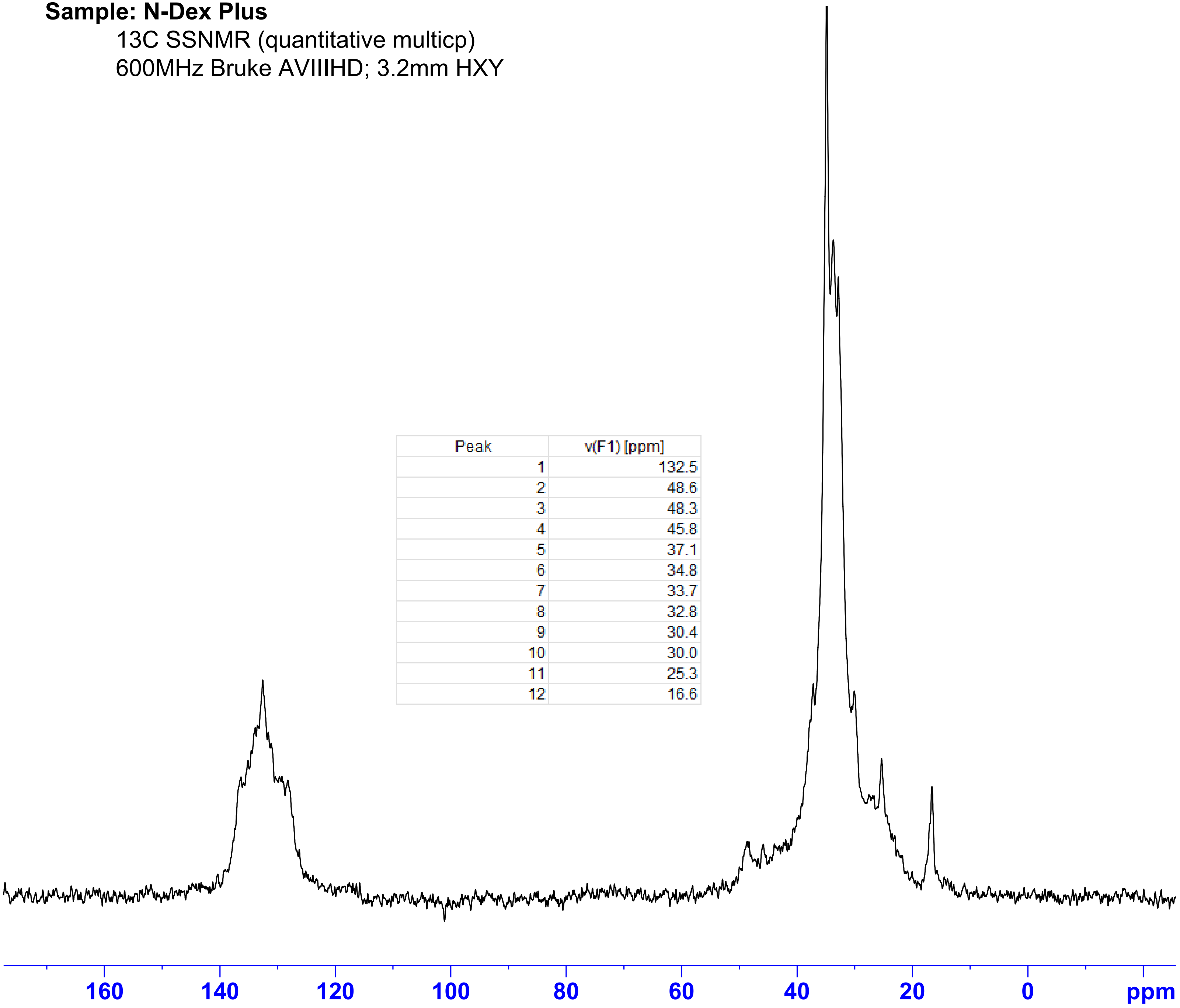

Peak	ν(F1) [ppm]
1	132.5
2	48.6
3	48.3
4	45.8
5	37.1
6	34.8
7	33.7
8	32.8
9	30.4
10	30.0
11	25.3
12	16.6

```
Current Data Parameters
NAME        HXY_13C_Vodovotz
EXPNO                      4
PROCNO                     1

F2 - Acquisition Parameters
Date_               20230411
Time                 12.35 h
INSTRUM                spect
PROBHD      H127000_0013 (
PULPROG         multicp_tlw
TD                      4096
SOLVENT
NS                     10240
DS                         8
SWH            59523.809 Hz
FIDRES         29.064360 Hz
AQ            0.0344064 sec
RG                    191.56
DW                 8.400 usec
DE                  6.50 usec
TE                       0 K
D1            3.00000000 sec
D3            0.00003400 sec
D11           0.40000001 sec
D13           0.00004400 sec
L0                         9
L31                    20000
ZGOPTNS
SFO1         150.9489952 MHz
NUC1                     13C
P1                  5.00 usec
P2                 10.00 usec
P5                100.00 usec
P6                100.00 usec
PLW1         43.00000000 W
PLW11        43.00000000 W
SFO2         600.2559261 MHz
NUC2                      1H
CPDPRG[2         spinal64_13
P3                  2.50 usec
PCPD2               4.80 usec
PLW2        122.00000000 W
PLW3         88.00000000 W
PLW13       122.00000000 W
am2           90 %

F2 - Processing parameters
SI                     16384
SF           150.9339227 MHz
WDW                       EM
SSB                        0
LB                  20.00 Hz
GB                         0
PC                      0.20
```

Sample: Pure Nitrile

13C SSNMR (quantitative multicp)

600MHz Bruke AVIIIHD; 3.2mm HXY

Peak	ν(F1) [ppm]
1	136.6
2	135.3
3	134.0
4	132.5
5	131.7
6	129.3
7	128.2
8	48.2
9	37.6
10	30.0
11	27.6
12	25.3
13	24.2
14	21.7
15	16.8

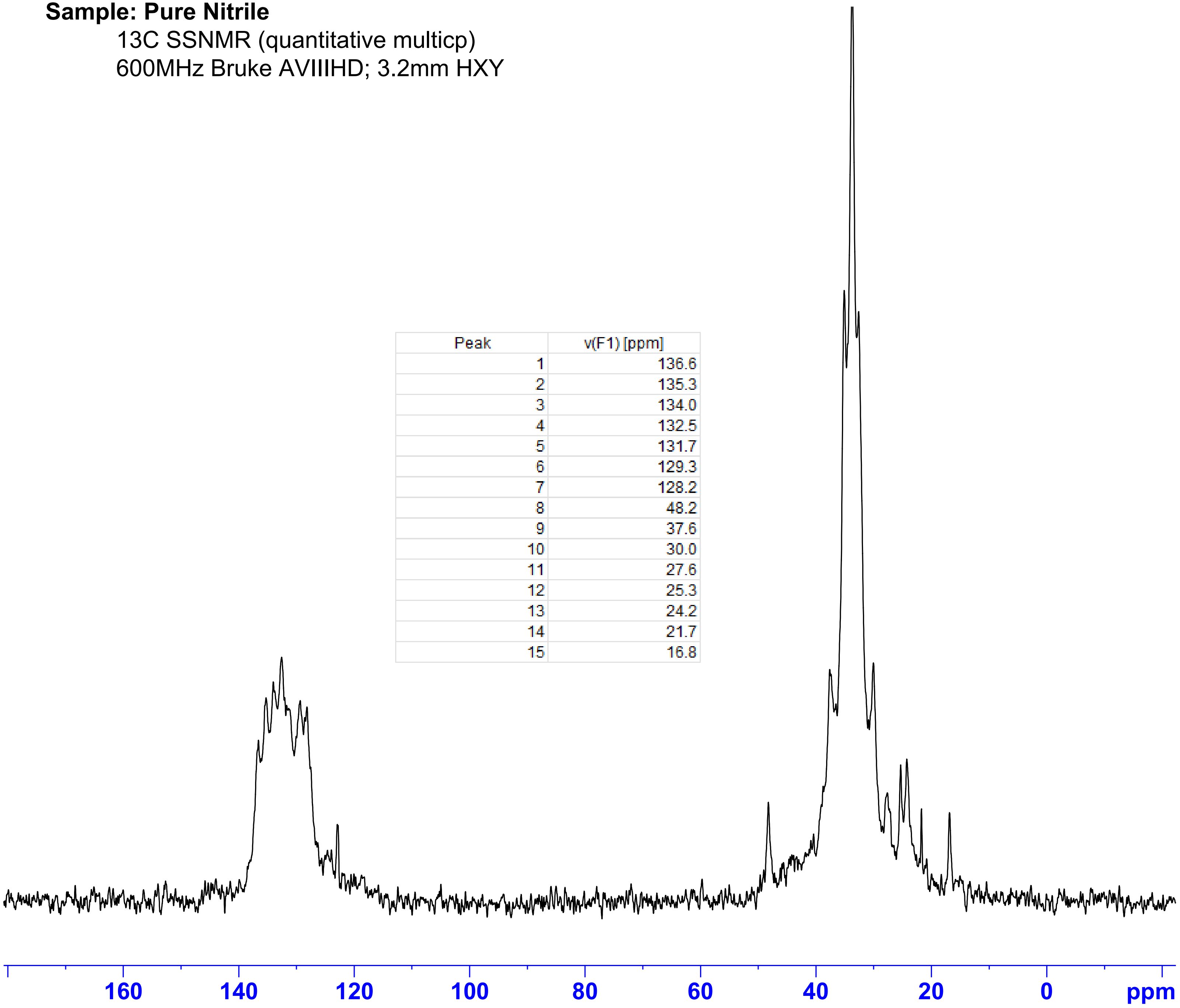


```
Current Data Parameters
NAME      HXY_13C_Vodovotz
EXPNO                    1
PROCNO                   1

F2 - Acquisition Parameters
Date_             20230406
Time                 10.08 h
INSTRUM              spect
PROBHD    H127000_0013 (
PULPROG        multicp_tlw
TD                    4096
SOLVENT
NS                   10240
DS                       8
SWH              59523.809 Hz
FIDRES           29.064360 Hz
AQ               0.0344064 sec
RG                  191.56
DW                   8.400 usec
DE                    6.50 usec
TE                       0 K
D1              3.00000000 sec
D3              0.00003400 sec
D11             0.40000001 sec
D13             0.00004400 sec
L0                       9
L31                  20000
ZGOPTNS
SFO1           150.9489952 MHz
NUC1                   13C
P1                    5.00 usec
P2                   10.00 usec
P5                  100.00 usec
P6                  100.00 usec
PLW1           43.00000000 W
PLW11          43.00000000 W
SFO2           600.2559261 MHz
NUC2                    1H
CPDPRG[2        spinal64_13
P3                    2.50 usec
PCPD2                 4.80 usec
PLW2          122.00000000 W
PLW3           88.00000000 W
PLW13         122.00000000 W
am2       90 %

F2 - Processing parameters
SI                   16384
SF             150.9339227 MHz
WDW                     EM
SSB                      0
LB                   20.00 Hz
GB                       0
PC                    0.20
```

Sample: Restore Touch

13C SSNMR (quantitative multicp)

600MHz Bruke AVIIIHD; 3.2mm HXY

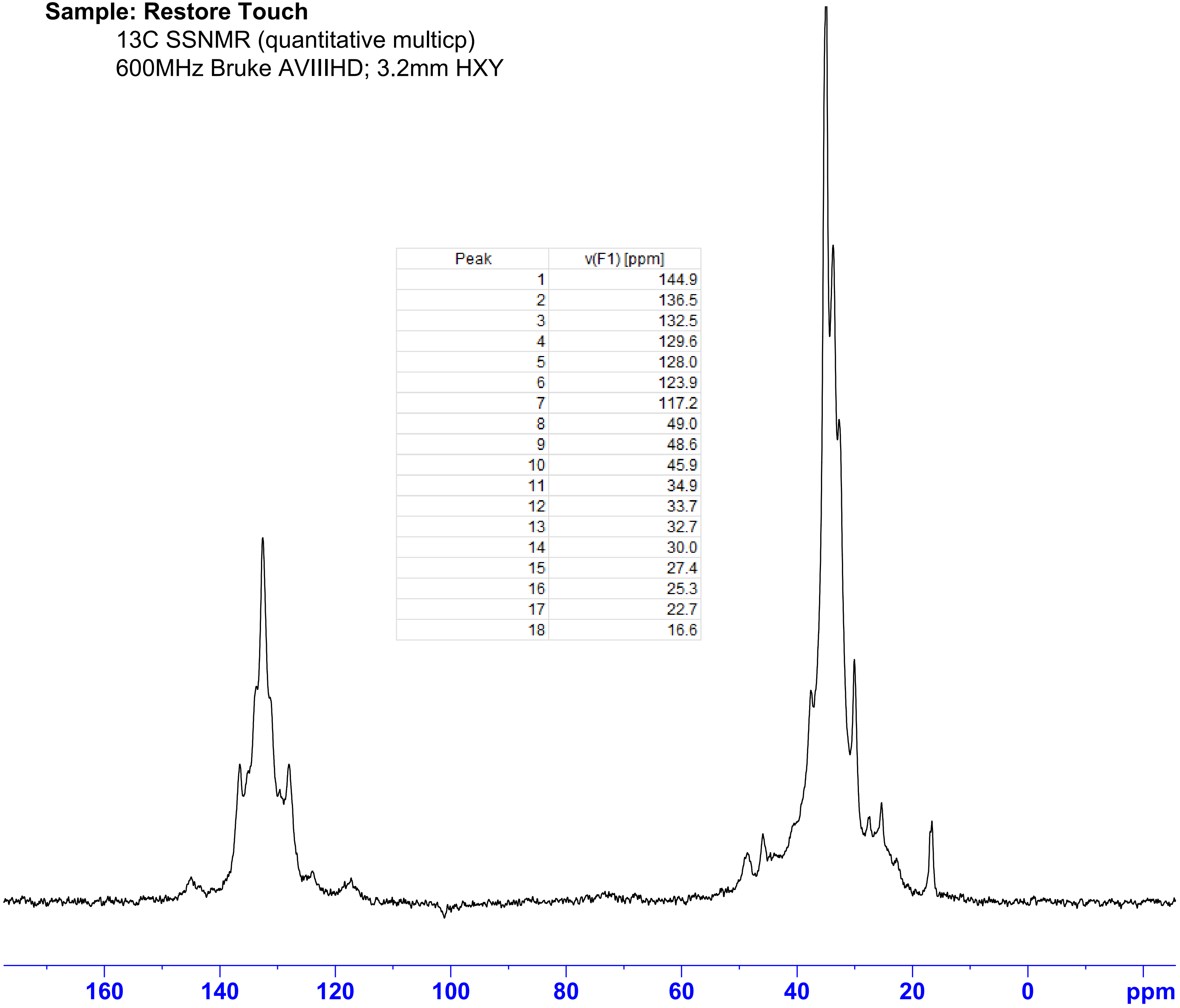

Peak	ν(F1) [ppm]
1	144.9
2	136.5
3	132.5
4	129.6
5	128.0
6	123.9
7	117.2
8	49.0
9	48.6
10	45.9
11	34.9
12	33.7
13	32.7
14	30.0
15	27.4
16	25.3
17	22.7
18	16.6

```
Current Data Parameters
NAME      HXY_13C_Vodovotz
EXPNO                    5
PROCNO                   1

F2 - Acquisition Parameters
Date_             20230412
Time                  9.31 h
INSTRUM              spect
PROBHD    H127000_0013 (
PULPROG       multicp_tlw
TD                    4096
SOLVENT
NS                   10240
DS                       8
SWH              59523.809 Hz
FIDRES           29.064360 Hz
AQ               0.0344064 sec
RG                  191.56
DW                   8.400 usec
DE                    6.50 usec
TE                       0 K
D1              3.00000000 sec
D3              0.00003400 sec
D11             0.40000001 sec
D13             0.00004400 sec
L0                       9
L31                  20000
ZGOPTNS
SFO1           150.9489952 MHz
NUC1                   13C
P1                    5.00 usec
P2                   10.00 usec
P5                  100.00 usec
P6                  100.00 usec
PLW1           43.00000000 W
PLW11          43.00000000 W
SFO2           600.2559261 MHz
NUC2                    1H
CPDPRG[2       spinal64_13
P3                    2.50 usec
PCPD2                 4.80 usec
PLW2          122.00000000 W
PLW3           88.00000000 W
PLW13         122.00000000 W
am2       90 %

F2 - Processing parameters
SI                   16384
SF             150.9339227 MHz
WDW                     EM
SSB                      0
LB                   20.00 Hz
GB                       0
PC                    0.20
```

Sample: Saveco PVC

13C SSNMR (quantitative multicp)

600MHz Bruke AVIIIHD; 3.2mm HXY

Peak	ν(F1) [ppm]
1	167.1
2	136.6
3	132.7
4	131.7
5	69.6
6	59.4
7	43.1
8	39.6
9	36.1
10	28.9
11	22.4
12	16.7
13	13.7

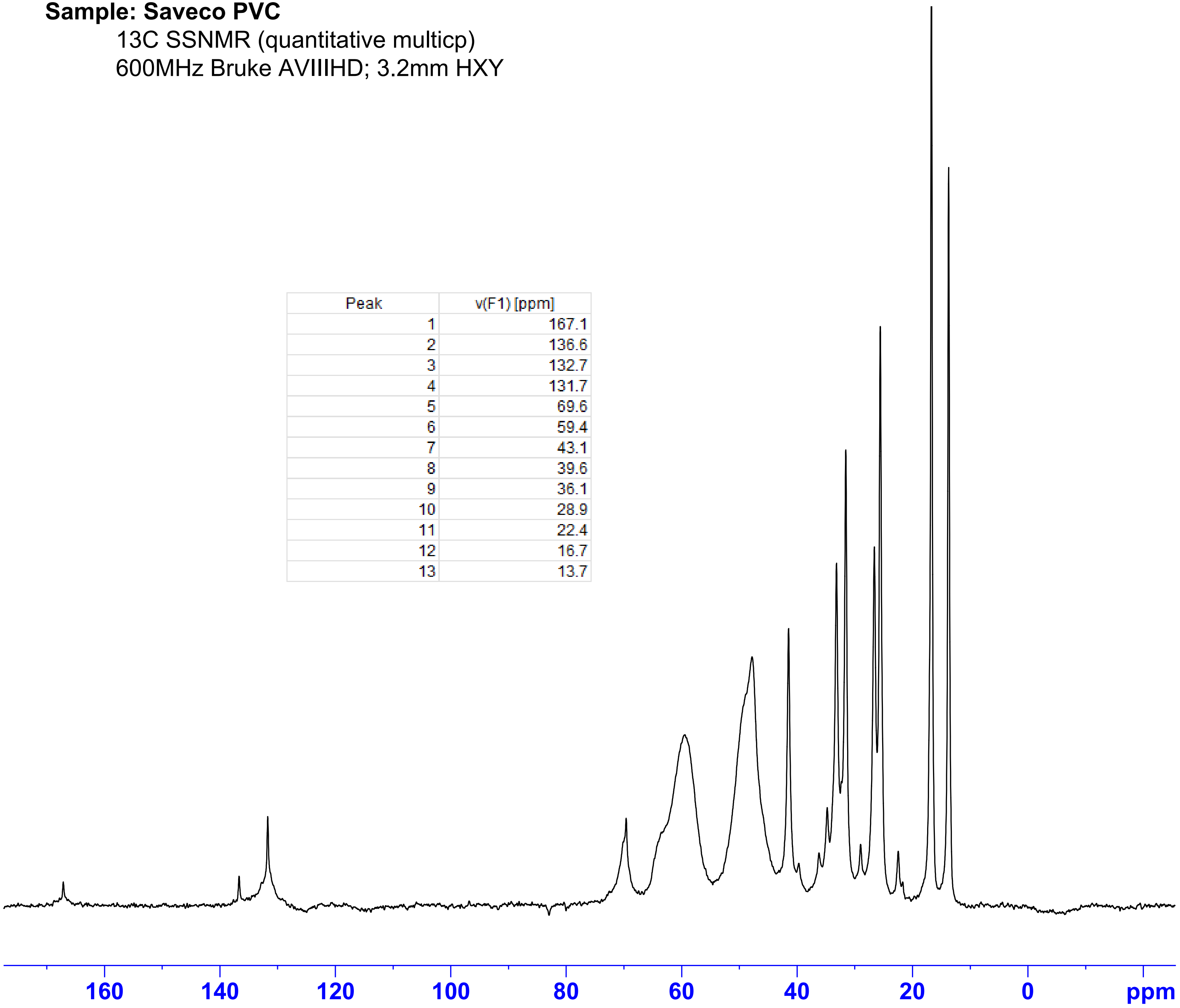


```
Current Data Parameters
NAME      HXY_13C_Vodovotz
EXPNO                    9
PROCNO                   1

F2 - Acquisition Parameters
Date_             20230506
Time                7.56 h
INSTRUM              spect
PROBHD    H127000_0013 (
PULPROG       multicp_tlw
TD                    4096
SOLVENT
NS                   10240
DS                       8
SWH              59523.809 Hz
FIDRES           29.064360 Hz
AQ               0.0344064 sec
RG                  191.56
DW                   8.400 usec
DE                    6.50 usec
TE                       0 K
D1              3.00000000 sec
D3              0.00003400 sec
D11             0.40000001 sec
D13             0.00004400 sec
L0                       9
L31                  20000
ZGOPTNS
SFO1           150.9489952 MHz
NUC1                   13C
P1                    5.00 usec
P2                   10.00 usec
P5                  100.00 usec
P6                  100.00 usec
PLW1           44.00000000 W
PLW11          44.00000000 W
SFO2           600.2555479 MHz
NUC2                    1H
CPDPRG[2       spinal64_13
P3                    2.50 usec
PCPD2                 4.80 usec
PLW2          112.00000000 W
PLW3           81.00000000 W
PLW13         112.00000000 W
am2       90 %

F2 - Processing parameters
SI                   16384
SF             150.9338167 MHz
WDW                     EM
SSB                      0
LB                   20.00 Hz
GB                       0
PC                    0.20
```

Sample: U.S. Medical

13C SSNMR (quantitative multicp)
600MHz Bruke AVIIIHD; 3.2mm HXY

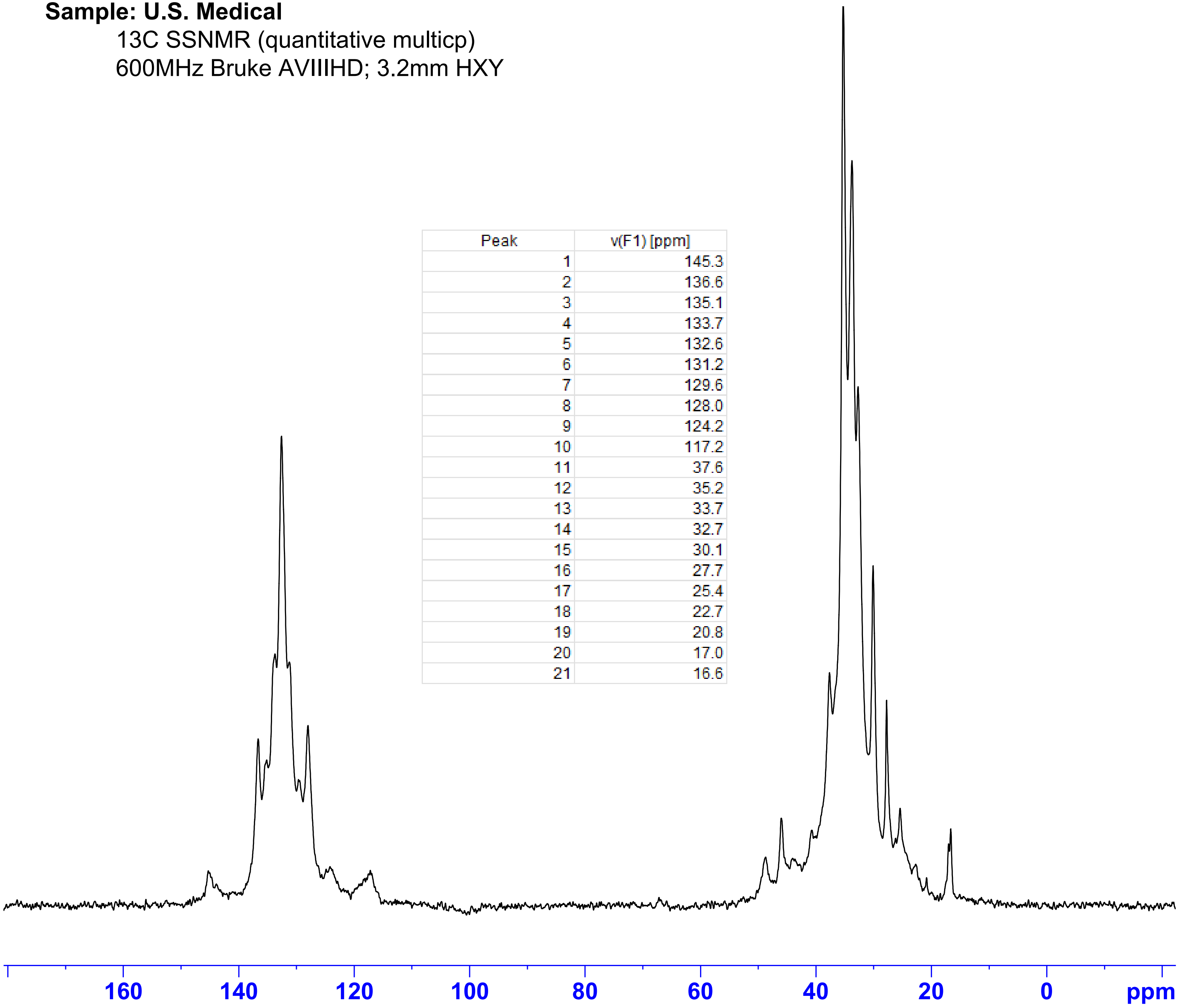

Peak	ν(F1) [ppm]
1	145.3
2	136.6
3	135.1
4	133.7
5	132.6
6	131.2
7	129.6
8	128.0
9	124.2
10	117.2
11	37.6
12	35.2
13	33.7
14	32.7
15	30.1
16	27.7
17	25.4
18	22.7
19	20.8
20	17.0
21	16.6

```
Current Data Parameters
NAME      HXY_13C_Vodovotz
EXPNO                    2
PROCNO                   1

F2 - Acquisition Parameters
Date_             20230407
Time                6.15 h
INSTRUM              spect
PROBHD    H127000_0013 (
PULPROG       multicp_tlw
TD                    4096
SOLVENT
NS                   10240
DS                       8
SWH              59523.809 Hz
FIDRES           29.064360 Hz
AQ               0.0344064 sec
RG                  191.56
DW                   8.400 usec
DE                    6.50 usec
TE                       0 K
D1              3.00000000 sec
D3              0.00003400 sec
D11             0.40000001 sec
D13             0.00004400 sec
L0                       9
L31                  20000
ZGOPTNS
SFO1           150.9489952 MHz
NUC1                   13C
P1                    5.00 usec
P2                   10.00 usec
P5                  100.00 usec
P6                  100.00 usec
PLW1           43.00000000 W
PLW11          43.00000000 W
SFO2           600.2559261 MHz
NUC2                    1H
CPDPRG[2       spinal64_13
P3                    2.50 usec
PCPD2                 4.80 usec
PLW2          122.00000000 W
PLW3           88.00000000 W
PLW13         122.00000000 W
am2       90 %

F2 - Processing parameters
SI                   16384
SF             150.9339227 MHz
WDW                     EM
SSB                      0
LB                   20.00 Hz
GB                       0
PC                    0.20
```

Sample: Vglove

13C SSNMR (quantitative multicp)

600MHz Bruke AVIIIHD; 3.2mm HXY

Peak	v(F1) [ppm]
1	175.4
2	171.7
3	167.9
4	167.3
5	136.7
6	131.8
7	75.6
8	69.8
9	68.0
10	66.8
11	59.3
12	47.6
13	45.8
14	41.4
15	33.1
16	31.5
17	30.2
18	26.6
19	25.5
20	21.6
21	16.6
22	16.3
23	13.6

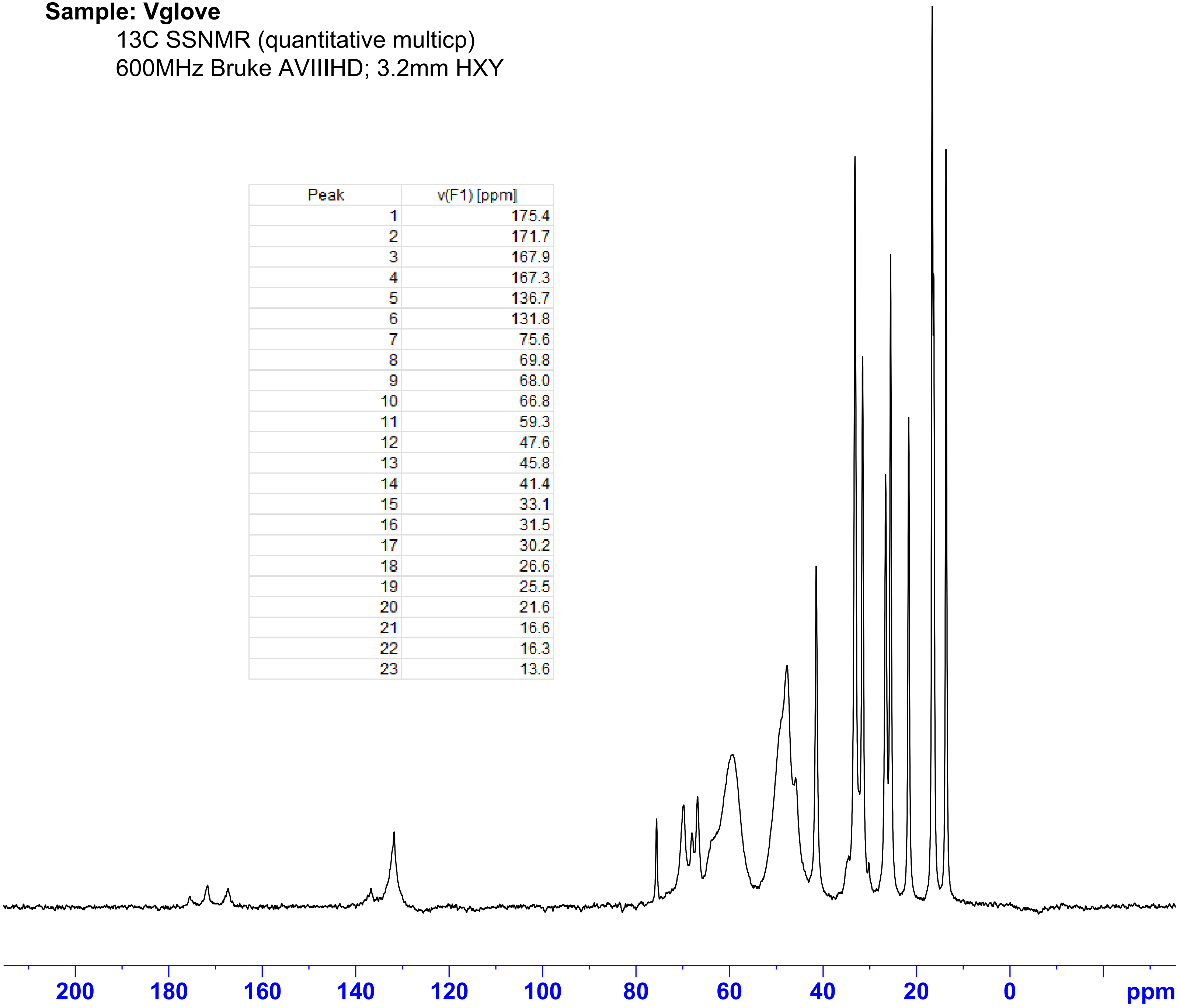

Current Data Parameters

NAME	HXY_13C_Vodovotz
EXPNO	3
PROCNO	1

F2 - Acquisition Parameters

Date_	20230408
Time	6.28 h
INSTRUM	spect
PROBHD	H127000_0013 (
PULPROG	multicp_tlw
TD	4096
SOLVENT	
NS	10240
DS	8
SWH	59523.809 Hz
FIDRES	29.064360 Hz
AQ	0.0344064 sec
RG	191.56
DW	8.400 usec
DE	6.50 usec
TE	0 K
D1	3.00000000 sec
D3	0.00003400 sec
D11	0.40000001 sec
D13	0.00004400 sec
L0	9
L31	20000
ZGOPTNS	
SFO1	150.9489952 MHz
NUC1	13C
P1	5.00 usec
P2	10.00 usec
P5	100.00 usec
P6	100.00 usec
PLW1	43.00000000 W
PLW11	43.00000000 W
SFO2	600.2559261 MHz
NUC2	1H
CPDPRG[2	spinal64_13
P3	2.50 usec
PCPD2	4.80 usec
PLW2	122.00000000 W
PLW3	88.00000000 W
PLW13	122.00000000 W
am2	90 %

F2 - Processing parameters

SI	16384
SF	150.9339227 MHz
WDW	EM
SSB	0
LB	20.00 Hz
GB	0
PC	0.20
