# Supplementary figures and images for: Nitrile glove composition and performance—Substandard properties and inaccurate packaging information
Source: PLoS One. 2024 Oct 31;19(10):e0312891. doi: 10.1371/journal.pone.0312891 (PMC11527158; doi:10.1371/journal.pone.0312891)

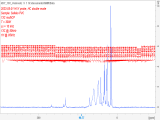

Supplement: S2 File — (ZIP) [file pone.0312891.s002.zip › ssnmr herkins /9/pdata/1/thumb.png]

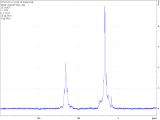

Supplement: S2 File — (ZIP) [file pone.0312891.s002.zip › ssnmr herkins /7/pdata/1/thumb.png]

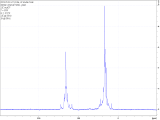

Supplement: S2 File — (ZIP) [file pone.0312891.s002.zip › ssnmr herkins /6/pdata/1/thumb.png]

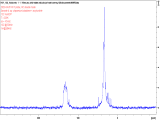

Supplement: S2 File — (ZIP) [file pone.0312891.s002.zip › ssnmr herkins /1/pdata/1/thumb.png]

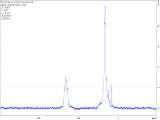

Supplement: S2 File — (ZIP) [file pone.0312891.s002.zip › ssnmr herkins /8/pdata/1/thumb.png]

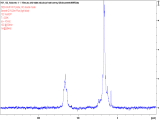

Supplement: S2 File — (ZIP) [file pone.0312891.s002.zip › ssnmr herkins /4/pdata/1/thumb.png]

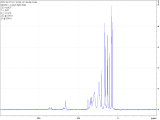

Supplement: S2 File — (ZIP) [file pone.0312891.s002.zip › ssnmr herkins /3/pdata/1/thumb.png]
